# Scalp Micro-Pigmentation via Transcutaneous Implantation of Flexible Tissue Interlocking Biodegradable Microneedles

**DOI:** 10.3390/pharmaceutics11110549

**Published:** 2019-10-23

**Authors:** Shayan Fakhraei Lahiji, Daniel Junmin Um, Youseong Kim, Jeesu Jang, Huisuk Yang, Hyungil Jung

**Affiliations:** 1 Department of Biotechnology, Building 123, Yonsei University, 50 Yonsei-ro, Seodaemun-gu, Seoul 03722, Korea; 2 Juvic Biotech, Inc., No. 208, Digital-ro 272, Guro-gu, Seoul 08389, Korea

**Keywords:** micro-pigmentation, scalp camouflage, flexible microneedle array, transdermal implantation, tissue interlocking microneedle

## Abstract

Alopecia, characterized by hair follicle blockage and hair loss, disrupts the normal cycle of hair growth. Although not a life-threatening condition, a growing body of evidence suggests that the psychological state of individuals experiencing alopecia can be highly influenced. Despite considerable research on hair loss treatment, interest in micro-pigmentation has increased in recent decades. Micro-pigmentation is an effective method to camouflage the visual contrast between the scalp and hair strands. However, the localization, intensity and dimension of microdots depend highly upon the physician performing the implantation. Incorrectly localized microdots within the skin may lead to patchy or faded micro-pigmentation. To overcome the limitations of conventional micro-pigmentation, we aimed to develop micro-pigment-encapsulated biodegradable microneedles (PBMs), capable of accurately implanting pigments below the epithelial-dermal junction of the scalp in a minimally invasive manner. A tissue interlocking microneedle technique was utilized to fabricate double-layered PBMs over a biodegradable flexible sheet, which could be washed off post-implantation. We confirmed that the intensity, dimension and insertion depth of 1000 μm-long PBMs was maintained on pig cadaver skin over time. This study suggested that the developed PBMs would serve as an attractive platform for scalp micro-pigmentation in the future.

## 1. Introduction

The hair growth cycle consists of three main phases: Growth (anagen), transition (catagen) and rest (telogen) [1,2]. The characteristics of the hair growth cycle in men and women can be evolved or disrupted in response to various environmental and genetic factors, including the conversion of testosterone to dihydrotestosterone (DHT), a primary androgen responsible for hair loss [3,4,5]. The lightly pigmented thin hairs are replaced by darker and thicker hairs in the beard during puberty, whereas the thick hairs of the scalp can be changed into thin scalp hairs over time, which, combined with hair loss, is also known as alopecia [6,7]. Androgenetic alopecia, telogen effluvium and alopecia areata are the most common types of hair loss, affecting millions of men and women around the world [8,9]. In androgenetic alopecia, an upregulation of androgen levels within hair follicles results in a short anagen phase and a faster anagen-telogen transition, leading to shortened hairs and the permanent blockage of hair follicles [10]. Patients with telogen effluvium experience a premature excessive shedding of hair strands, which increases the risk of psychological trauma [11]. 

Alopecia areata, on the other hand, is an autoimmune disorder in which hair follicles enter into a dystrophic catagen phase, increasing the risk of permanent hair follicle loss [12].

Although the disruption of the hair cycle is not life-threatening, it can have major effects on the psychological state of individuals [13,14,15]. Therefore, various solutions, including United States Food and Drug Administration (FDA)-approved, DHT-reducing medications and hair grafting surgeries, were introduced to treat alopecia [16]. DHT-reducing agents, such as Finasteride and Minoxidil, are prescribed to prevent further hair thinning [17,18]. However, any discontinuation of medication brings back the normal rate of hair loss [19], and these medications neither restore the lost hair, nor reverse baldness, constraining patients with progressive alopecia to seek hair transplants [20]. Hair grafting, while effective, is an invasive procedure, resulting in a large permanent scar upon the scalp. Moreover, as most patients undergoing hair transplantation suffer from progressive androgenic alopecia, hair loss still continues post-surgery. 

Scalp micro-pigmentation is a camouflaging approach that reduces the visual contrast between the scalp and the hair strands [21]. In this method, pigments in the form of microdots are implanted below the epithelial-dermal junction and last between 1 to 2 years [22]. Briefly, at 1 month post-implantation, the basement membrane layer of the epidermis fully reforms, trapping the pigmented fibroblasts within the network of connective tissues [23]. Micro-pigmentation is advantageous because it provides a quick, effective and efficient solution for patients suffering from hair loss [24]. Moreover, it is safe, patient-friendly and economical, compared with hair grafting surgery, because it does not involve an invasive procedure. The localization, intensity and size of the microdots, however, depends on the physician performing the implantation [22]. As the microdots diffuse over time, an incorrect implantation depth or volume might result in an irrevocable patchy appearance over the scalp. Thus, the development of a micro-pigmentation platform that is capable of precise pigment implantation in a minimally invasive manner would overcome the limitations of current hypodermic needle-based scalp micro-pigmentation. 

In the present study, we report the development of micro-pigment encapsulated biodegradable microneedles (PBMs) as a novel micro-pigmentation platform to implant an exact dose of micro-pigment at the targeted skin depth. PBMs comprise a micro-dimensioned polymeric transdermal system designed to implant the encapsulated micro-pigments in a minimally invasive manner [25,26,27]. A recently developed fabrication technique that produces a “tissue interlocking dissolving microneedle”, capable of the accurate delivery of encapsulated materials, is employed to achieve a precise implantation of micro-pigments below the epidermis [28]. PBMs comprise an unpigmented base layer, acting as a shaft to implant micro-pigments beneath the epidermis, and a micro-pigment encapsulating top layer. Through a series of ex vivo and in vivo evaluations, we systematically demonstrate that PBMs are capable of an accurate implantation of micro-pigments at the target dermal depth in a minimally invasive manner. In addition, by assessing the cutaneous permeation of micro-pigments implanted at different dermal depths, we show that the intensity and dimension of 1000 μm-long, PBM-implanted microdots can be maintained within pig cadaver skin, which has similar thickness and structural properties to human skin. 

## 2. Materials and Methods 

### 2.1. Fabrication of PBMs

Solutions were prepared by mixing hyaluronic acid (HA, 32 kDa, Soliance, Pomacle, France) with distilled water and a safety-approved black tattoo ink (INT019-1, Intenze, Rochelle Park, NJ, USA). Micro-pigment-encapsulated biodegradable microneedles (PBMs) examined in ex vivo evaluations, were fabricated at two heights: 800 ± 45 μm and 1000 ± 51 μm, using a centrifugal lithography dissolving microneedle fabrication technique. First, a thin flexible layer of biodegradable sheet was fabricated by coating 20% HA (*w*/*w*) over a metal plate. The unpigmented HA droplet was then dispensed (SHOT mini 100-s, Musashi, Tokyo, Japan) in 5 × 5 arrays over the flexible sheet, positioned 200 μm and 400 μm away from a non-sticky plate, and centrifuged at 300× *g* for 1 min (Combi centrifuge, Hanil, Gimpo, Korea). 

The non-sticky plate was then removed and the resulting 200 μm- and 400 μm-long wine-glass-shaped primary layers were solidified for another 5 min. Subsequently, micro-pigment encapsulated polymer droplets were dispensed on top of the solidified primary layers and centrifuged therewith at 500× *g* for 1 min. The height of the micro-pigment-encapsulated layer fabricated over 200 μm and 400 μm base layers was fixed at 600 ± 48 μm. Thus, the final height of the PBMs fabricated over 200 μm and 400 μm unpigmented layers, were 800 ± 45 μm and 1000 ± 51 μm, respectively. PBMs employed for in vivo evaluations were fabricated using the same method at a total height of 400 ± 26 μm on a 100 μm-long unpigmented base layer.

### 2.2. Viscosity Measurements 

The viscosities of pigment diluted in distilled water (25%, 50%, 75% and 100%) mixed with HA in powder form (20%, 40%, 60% and 80%) were measured using a Merlin VR viscometer (Rheosys, Plainsboro, NJ, USA) equipped with a 30 mm parallel plate at a shear rate of 300 1/s. Before evaluation, all polymer solutions were stirred for 5 min to achieve a homogeneous mixture. The temperature of the viscometer plate was set at 25 °C throughout the experiment to avoid any solidification of the samples. 

### 2.3. Flow Cytometry Analysis

Human dermal papilla cells (Cell Applications, San Diego, CA, USA) were cultured in Dulbecco’s modified Eagle’s medium (DMEM). The cells were then treated separately with pigment, HA and a mixture of pigment-HA for 24 h. The biocompatibility of the mixtures was measured using fluorescence-activated cell sorting (FACS, Aria II, BD Biosciences, Franklin Lakes, NJ, USA) by assessing the state of the cells post-treatment. Briefly, cells were dissociated into single cells using trypsin-EDTA (Gibco, Grand Island, NY, USA) and incubated with fluorescent-labeled antibodies: Anti-AnnexinV-fluorescein isothiocyanate (FITC) and anti-propidium iodide (eBioscience, San Diego, CA, USA) for 10 min. The results were analyzed using the FlowJo software (BD Biosciences).

### 2.4. Fracture Force and Skin Penetration Force Assessment

The mechanical fracture force of the 800 μm- and 1000 μm-long PBMs was measured using a material force analyzer (Z0.5TN, Zwick/Roell, Ulm, Germany) at a speed of 1 mm/min. PBMs were positioned against a sensor probe, which applied pressure on the tip portion of PBMs and measured their fracture force (n = 10). The skin insertion force was measured by positioning PBMs on the sensor probe and inserting PBMs at a speed of 1 mm/min into pig cadaver skin (CRONEX, Seoul, South Korea) with a surface area 1 cm^2^ and a thickness of 2 mm (*n* = 10). 

The successful skin penetration rate of the PBMs was calculated by counting the number of detectable pierced spots (*n* = 5 arrays) at 30 min post-implantation using the M165 FC bright-field optical microscope (Leica, Wetzlar, Germany). 

### 2.5. Ex Vivo Micro-Pigment Implantation Analysis

The permeation and dissolution characteristic of the 800 μm- and 1000 μm-long PBMs were evaluated using a Franz cell diffusion system (Hanson, Los Angeles, CA, USA) up to 28 days (*n* = 4/group). The temperature of the Franz cell chamber was set at 35 ± 1 °C and stirred at 250 rpm to mimic the body’s blood circulation. The fully-dissolved PBMs were gently washed off the skin at 30 min post-implantation. The skin surface was recorded at days 1, 7 and 28 under a bright-field optical microscope. Subsequently, the treated skins were separately sectioned to evaluate the permeation pattern of the micro-pigment across the dermis layer. 

The pigmentation regions of PBMs over the skin were quantified using ImageJ software (National Institutes of Health, Bethesda, MD, USA) in two categories of (i) the main pigmentation area, being the central region of microdots with the highest pigment intensity (90 to 100%), and (ii) the diffusion ring, which is the faded pigment with lower intensity (10 to 90%) surrounding the microdots (Figure S1). 

To quantify the microdot area, the dimension of the micro-pigment layer in PBMs (250 ± 25 μm) was set at 1, and the relative pigmentation area was calculated accordingly. The relative diffusion ring area was measured separately on each day, based on the mean pigmentation area set at 1 and quantified using ImageJ accordingly. 

### 2.6. In Vivo Assessment

Male BALB/c mice (8 weeks old) were purchased from YoungBio (Gyeonggi-do, Korea) and given one week to adapt to their new environment. The dorsal skin of the mice was shaved using a clipper and left for 1 day to avoid skin damage. Mice were then treated separately with a control and 400 ± 26 μm-long PBMs for 28 days (*n* = 4/group). Throughout the experiment, the mice were maintained under a 12-h light/dark cycle with ad libitum access to food and water. 

Transepidermal water loss (TEWL) assessments were conducted in the control group and the PBM-implanted group using AquaFlux™ (Biox Systems, London, UK). Briefly, PBMs were implanted into the skin for 30 min to fully dissolve, followed by the removal of the flexible sheet. The TEWL readings were taken at 0, 1, 2, 3, 4, 5, 6, 12 and 24 h post implantation (*n* = 4/group). 

All procedures were performed in accordance with the guidelines and regulations of the experimentation ethics by Yonsei Laboratory Animal Research Center (YLARC), with approval from the “International Animal Care and Use Committee”on Augest 2019, registered as IACUC-A-201907-937-02.

### 2.7. Statistical Analysis

Means (± the standard error of the mean (SEM)) were compared using Student’s *t*-test or one-way Analysis of Variance (ANOVA) using GraphPad Prism 6 software (GraphPad Software, Inc., La Jolla, CA, USA). P-values of < 0.05 were considered statistically significant.

## 3. Results and Discussion

### 3.1. Fabrication of PBMs

**Figure 1:**
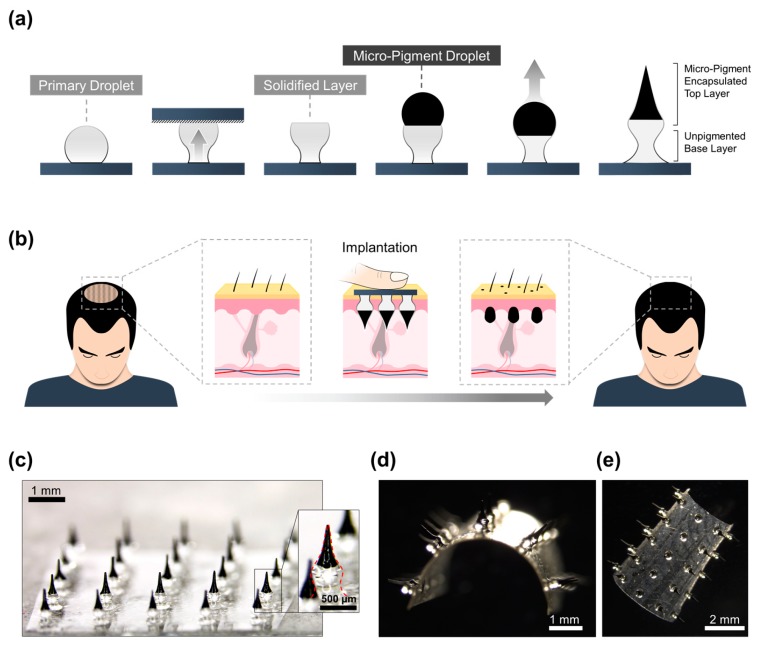
Fabrication and implantation of micro-pigment-encapsulated biodegradable microneedles (PBMs). (**a**) PBMs are fabricated in two phases of (i) unpigmented primary layer, and (ii) micro-pigment-encapsulated secondary layer. Briefly, the unpigmented droplet is dispensed over a thin hyaluronic acid (HA) sheet and centrifuged to form a wine glass-shaped primary layer. The micro-pigment-encapsulated droplet is then dispensed over the solidified primary layer and centrifuged to form PBMs. (**b**) Upon complete implantation, PBMs reside in the dermis layer and reduce the contrast between scalp and hairs. (**c**) A microscopic image of a 5 × 5 PBM array. (**d**) PBMs are fabricated over flexible biodegradable sheets that can be washed off post-implantation. (**e**) Top view of the bent PBM-fabricated flexible sheet.

The precise implantation of micro-pigments requires a highly accurate implantation system. Thus, the tissue interlocking microneedle fabrication method, a highly precise transdermal delivery system, was used to fabricate these PBMs. Hyaluronic acid (HA), a natural glycosaminoglycan widely used to boost the elasticity and hydration of the skin, was employed as the backbone matrix of the PBMs [29]. Briefly, fabrication of PBMs consisted of two main phases of (i) unpigmented base layer and (ii) a micro-pigment-encapsulated top layer fabrication. First, the unpigmented HA droplet was dispensed over a flexible sheet and transformed into a solidified wine glass-shaped layer using centrifugal force. The unpigmented primary layer acts as a shaft, implanting the micro-pigments at the targeted skin depth. Subsequently, the micro-pigment-encapsulated HA droplet was dispensed over the solidified primary base layer. Centrifugation of the secondary droplet created a double-layered PBM with a narrow neck, a wide mid-portion, and a sharp tip (Figure 1a). PBMs, because of their morphology, are capable of achieving a precise implantation of micro-pigments without utilizing an applicator [28]. Thus, individuals can use finger force to apply PBMs to the targeted scalp region and achieve a highly accurate implantation (Figure 1b).

To investigate the permeation and diffusion characteristics of the PBMs, they were fabricated in 5 × 5 arrays with a 1.5 mm pitch between each PBM (Figure 1c). According to their race, gender and age, individuals have different dermal thicknesses; therefore, the volume and height of the micro-pigment-encapsulated layer can be optimized by adjusting the pigmented droplet volume and the height of the primary base layer. In addition, based on an individual’s hair density and hair growth pattern, PBMs can be fabricated in random arrangements to provide the most realistic visual appearance (Figure S2). The scalp is not a flat surface; therefore, fabricating PBMs over a rigid base might have resulted in the unequal insertion of PBMs [30]. Therefore, by fabricating PBMs over a thin flexible sheet, the implantation accuracy of the arrays was further enhanced (Figure 1d,e). The flexible polymeric sheet is made of biodegradable HA, which can be simply washed off after the complete dissolution of the PBMs. 

### 3.2. Optimization of PBMs

**Figure 2:**
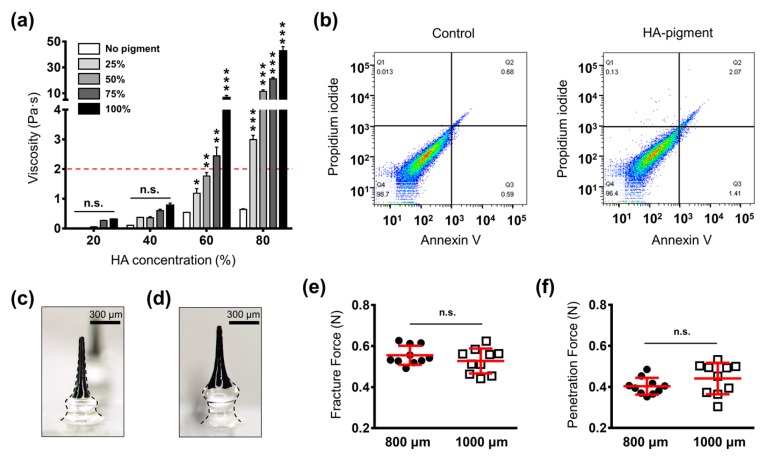
Optimization of micro-pigment-encapsulated biodegradable microneedle (PBM) fabrication parameters. (**a**) Evaluation of hyaluronic acid (HA)-pigment mixture viscosity. Increasing the HA and pigment concentration elevated the viscosity of the mixture. The dashed red line indicates the maximum viscosity that can be employed in the fabrication of PBMs through centrifugal lithography. (**b**) Fluorescence-activated cell sorting (FACS) results indicated that the PBMs did not stimulate apoptosis in human dermal papilla cells. (**c**) Microscopic images of a single PBM fabricated at 800 μm and (**d**) 1000 μm. Regardless of the height of the unpigmented base layer, the micro-pigment-encapsulated top layer had the same geometry in both PBMs. (**e**) Fracture force analysis of PBMs confirmed their capability to penetrate the skin without breakage. (**f**) PBMs successfully penetrated the skin upon applying a minimum force of 0.40 ± 0.01 N (800 μm) and 0.44 ± 0.02 (1000 μm). Data in (**a**,**e**,**f**) are expressed as the mean ± SEM. * *p* < 0.05, ** *p* < 0.01, and *** *p* < 0.001.

PBMs were fabricated using the centrifugal lithography method in which the viscosity of the backbone polymer must not exceed 2 Pa·s [31]. The concentration of the backbone polymer correlates with the mechanical strength of the PBMs; therefore, it is necessary to select the highest concentration of HA [32,33]. We found that there was no significant difference in the visual intensities of 25%, 50%, 75% and 100% pigment mixtures. Thus, to probe the viscosity of the mixture, HA at 20%, 40%, 60% and 80% was mixed with pigment at 25%, 50%, 75% and 100% concentrations. The results indicated that by increasing the concentration of HA and the pigment, the viscosity increased (Figure 2a). The impact of pigment addition upon viscosity was more dramatic in mixtures with higher concentrations of HA (60% and 80%). Among the assessed groups, HA 60% with pigment 50% at 1.76 ± 0.11 Pa·s showed the highest HA and pigment concentrations within the possible PBM fabrication range. Therefore, this mixture was selected as the optimal combination for the fabrication of PBMs.

Next, to evaluate the biocompatibility of PBMs, we performed a fluorescence-activated cell sorting (FACS) analysis, which allowed us to identify cells in the normal (−/−), early apoptotic (+/−), and late apoptotic (+/+) state. The results indicated that HA, pigment and the HA-pigment mixture employed in the fabrication of the PBMs maintained 97.0%, 96.9% and 96.4% of viable cells, respectively (Figure 2b, Figure S3). Hence, we concluded that the PBMs are biocompatible and safe for transdermal implantation. 

In conventional micro-pigmentation, the pigment is generally implanted at 1 mm deep inside the skin; therefore, after a series of skin regenerations and tissue shedding, it becomes located below the epithelial-dermal junction [22]. 

PBMs are dissolved inside the skin; therefore, their diffusion and permeation characteristics must be evaluated at different implantation depths. To find the optimal implantation depth for the PBMs, which would maintain their visual intensity over time, and does not form a patchy appearance, we fabricated them in two heights of 800 ± 45 μm and 1000 ± 51 μm, with unpigmented base layers of 200 μm and 400 μm, respectively (Figure 2c,d). The tip dimension, height and volume of the micro-pigment-encapsulated top layer were fixed at 34 ± 11 μm, 600 ± 48 μm and 0.41 ± 0.05 mg, respectively. Fixing the geometry of the micro-pigment layer allowed us to accurately assess the impact of changing the insertion depth on the visual intensity of the implanted PBMs. Moreover, to avoid any accidental detachment of the PBMs post-application, the mid-portion (300 ± 36 μm) was fabricated with a larger diameter compared to with that of the neck (250 ± 31 μm in 200 μm-long base and 200 ± 27 in 400 μm-long base). As shown previously, the narrow neck of tissue interlocking microneedles increased the tissue attachment force, thereby improving the implantation accuracy of the encapsulated agent [28].

### 3.3. Skin Insertion Analysis

Major limitations of hypodermic injection are the biohazardous wastes created post injection, pain and poor patient compliance [34]. Thus, using PBMs, in addition to the precise implantation of micro-pigments, we aimed to develop a minimally invasive approach that minimized pain levels. To ensure that both PBMs were capable of penetrating the skin without breakage, their mechanical fracture forces were measured. Both the 800 μm- and 1000 μm-long PBMs, at 0.55 ± 0.01 N and 0.52 ± 0.02 N, respectively, had the required mechanical strength to penetrate the skin (Figure 2e, Figure S4). Next, to evaluate the skin insertion force, PBMs were inserted into pig cadaver skin and the force at which penetration occurred was measured. The skin penetration forces for the 800 μm- and 1000 μm-long PBMs were 0.40 ± 0.01 N and 0.44 ± 0.02 N, respectively, with no significant difference between them (Figure 2f, Figure S5). The fact that the skin does not present the same thickness and structural properties throughout the body might have been responsible for the noticeable difference in the insertion forces [35]. A previous report comparing the pain levels of a 26-gauge hypodermic needle with microneedles in human subjects, confirmed that microneedles fabricated at heights of 700 μm and 960 μm (the closest ones to the PBMs evaluated in this study) evoked approximately 10% and 26% of the pain associated with the hypodermic needle [36]. Thus, we assumed that PBMs fabricated at 800 μm and 1000 μm would exhibit a low level of pain in individuals [37]. However, the detailed pain level evaluation of PBMs on human subjects remains to be performed in a future study.

### 3.4. Ex Vivo Penetration, Dissolution, and Permeation Assessment

**Figure 3:**
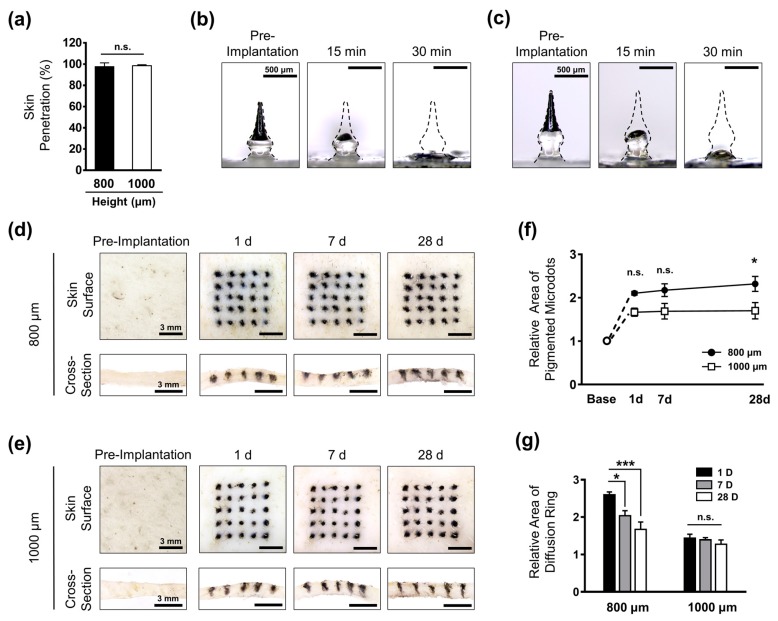
Transcutaneous analysis of micro-pigment-encapsulated biodegradable microneedles (PBMs). (**a**) Both 800 μm- and 1000 μm-long PBMs showed a high skin penetration success rate. (**b**) The micro-pigment-encapsulated top layer in both the 800 μm-long PBM and (**c**) 1000 μm-long PBMs were dissolved at 15 min post-implantation. At 30 min, the unpigmented primary layer was completely dissolved. (**d**) Implantation of 800 μm-long PBM into the pig cadaver skin resulted in the formation of a large diffusion ring on the skin surface (upper panels). The cross-section of skin confirmed the localization of the micro-pigment up to 28 days (lower panels). (**e**) The diffusion ring in the 1000 μm-long PBM-implanted skin was barely visible. The dimension of microdots was highly maintained up to 28 days (upper panels). Cross-section images revealed a sharper localization of micro-pigments compared with the 800 μm-long PBM-implanted skin. (**f**) Comparison of microdots confirmed the well-maintained micro-pigmentation in the 1000 μm-long PBM-implanted skin up to 28 days. (**e**) The diffusion ring was significantly reduced over time in the 800 μm-long PBM-implanted skin. Data in (**a**,**f**,**g**) are expressed as the mean ± SEM. * *p* < 0.05, and *** *p* < 0.001.

To achieve a precise implantation of micro-pigments inside the skin, the complete insertion and interlocking of the whole array plays a crucial role. Moreover, because each individual applies the force differently, the risk of incomplete insertion is high, leading to an uneven dissolution of encapsulated micro-pigments. In the present study, the PBMs were fabricated over a flexible sheet; therefore, we achieved remarkably high successful skin penetration rates of 97.60% ± 1.6% and 98.40% ± 2.19% for the 800 μm- and 1000 μm-long PBMs, respectively (Figure 3a). These findings confirmed that both groups were capable of being completely implanted as a 5 × 5 array of PBMs inside the skin. Next, to measure the dissolution time of PBMs, we employed Franz cell diffusion, a system that mimics the natural blood circulation of the human body. PBM-implanted pig cadaver skins were placed over the Franz cell diffusion system, and the morphological changes of PBMs were captured up to 30 min. The majority of the micro-pigment portion in both the 800 μm- and 1000 μm-long PBMs was dissolved at 15 min post-application (Figure 3b,c). At 30 min, the unpigmented base layer of the PBMs was almost completely dissolved, confirming the successful implantation of the micro-pigments inside the skin.

The transcutaneous dissolution and permeation characteristics of the PBMs were evaluated through implantation into a pig cadaver skin placed over the Franz cell diffusion system for 28 days. The initial intensity of the micro-pigment was maintained in both the 800 μm- and 1000 μm-long PBMs up to 28 days. In the 800 μm-long PBM-implanted skin, however, we observed a diffusion ring surrounding each application spot on day 1 and day 7. The dimensions of the microdots were further expanded up to 28 days post-application, with sharper edges and smaller diffusion rings compared with those on day 1 (Figure 3d). By contrast, in the 1000 μm-long PBM-implanted skin, the microdots were sharp and distinct, with a significantly smaller diffusion ring around the spots at day 1 (Figure 3e). Compared with that at day 1, at day 28, the dimension of the microdots was highly maintained, indicating localization of micro-pigments within the skin tissues. Cross-sectional images confirmed the localization of the micro-pigments within the tissues treated with both the 800 μm- and 1000 μm-long PBMs at 28 days post-implantation. In addition, the localization of the 1000 μm-long PBMs was sharper, with less diffusion compared with that of the 800 μm-long PBMs. Individuals with distinct dermal properties might display different permeation and dissolution patterns of PBMs implanted inside their skin. Therefore, performing a test implantation to evaluate the diffusion pattern of PBMs and optimizing them based on the individual’s skin characteristics would increase the overall effectiveness of PBM-based micro-pigmentation.

The microdots on the skin surface were quantified in two separate categories of (i) core pigmentation, and (ii) the diffusion ring. Core pigmentation refers to the region with the highest pigment intensity detected by the ImageJ software, whereas the diffusion ring is the surrounding area with a lower intensity. At day 1 post-implantation, the area of pigmented spots was expanded up to 2.11 ± 0.04 and 1.66 ± 0.09 for the 800 μm and 1000 μm-long PBMs, respectively (Figure 3f). At 28 days post-implantation, the pigmentation area was further increased up to 2.31 ± 0.17 in the 800 μm-long PBM implanted skin, whereas it was highly maintained in 1000 μm-long PBM implanted skin at 1.71 ± 0.18. 

These findings showed that deeper implantation of PBMs at 1000 μm leads to the precise localization of the micro-pigments and minimized diffusion over time. Furthermore, the continuous enlargement of the micro-pigment area in the 800 μm-long PBM-implanted skin may lead to a patchy appearance over time; therefore, the 1000 μm-long PBMs were expected to be the appropriate candidates for the fabrication of PBMs.

To examine the transcutaneous permeation characteristics of the PBMs, the dimensions of the diffusion rings surrounding the microdots were measured (Figure 3g). In the 800 μm-long PBM-implanted skin, the diffusion ring was extended up to 2.59 ± 0.07 at day 1, and was significantly reduced over time to 1.67 ± 0.19 at day 28. As expected, a markedly smaller diffusion ring was measured in the skin treated with the 1000 μm-long PBMs, at 1.44 ± 0.10 (day 1), which then reduced to 1.27 ± 0.11 at day 28. Consistent with the core pigmentation results, these findings confirmed that a deeper implantation of PBMs improved the localization of microdots and prevented the risk of over-diffusion up to 28 days post-implantation. It is important to consider the fact that since the experiments were performed ex vivo, the micro-pores were not healed post-implantation, which might have affected the absolute localization of PBMs within the skin tissues. Pig skin has similar structural properties, elasticity and thickness to human skin; therefore, these findings prompted us to conclude that PBMs fabricated at a height of 1000 μm were the optimal candidates for micro-pigmentation in human subjects.

### 3.5. Evaluation of in vivo Implantation Characteristics

**Figure 4:**
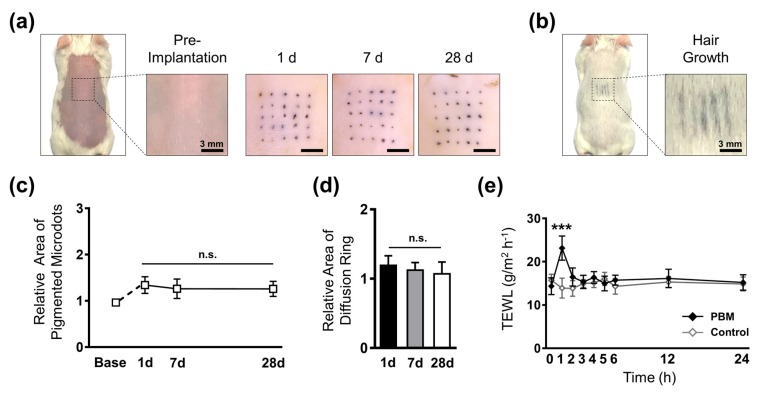
In vivo assessment of micro-pigment-encapsulated biodegradable microneedles (PBMs) in mice. (**a**) The dorsal skin of mice was shaved and implanted with PBMs. The visual intensity of the micro-pigments was maintained up to 28 days post-implantation. (**b**) Micro-pigments were visible on the dorsal skin after hair growth. (**c**) The dimension of microdots were expanded on day 1 and declined from day 7. (**d**) Diffusion rings surrounding the microdots were relatively small, with no significant alternations over time. (**e**) Transepidermal water loss (TEWL) analysis of mice at the implantation site confirmed that skin barrier functionality was restored at 2 h post-implantation with PBMs. Data in (**c**–**e**) are expressed as the mean ± SEM. * *p* < 0.05, and *** *p* < 0.001.

The closure of micro-wounds formed upon PBM implantation would affect the localization and intensity of the micro-pigments. Mice have a thinner dermis layer compared with that of pigs; therefore, we optimized the height of PBMs based on mouse skin thickness at 400 ± 26 μm comprised of a 100 μm-long unpigmented base layer. To probe the influence of wound healing and skin reepithelization on the micro-pigments, PBMs were implanted into the dorsal skin of mice and evaluated up to 28 days (Figure 4a). Comparison of the visual intensity of the microdots over the skin at day 1 and day 28 confirmed the localization of the micro-pigments beneath the epidermis. In contrast to the ex vivo results, a remarkably smaller core pigmentation and diffusion ring was observed. We assumed that the difference reflected the higher structural density of mouse skin compared with pig cadaver skin, which led to a concentrated localization of the micro-pigments. Furthermore, the in vivo findings confirmed that the visual intensity of the micro-pigments was well maintained, even after hair growth at the dorsal region (Figure 4b). 

Consequently, to quantify the size and pattern of the dissolution of the micro-pigments, we measured the area of each microdot along with their surrounding diffusion rings. Similar to the ex vivo pattern, the microdots were expanded up to 1.34 ± 0.18 (day 1), followed by a non-significant decline at day 7. The dimension of the microdots was then maintained at 1.25 ± 0.16 up to 28 days post-implantation (Figure 4c). Crucially, as the diffusion ring fades over time, to achieve a well-maintained micro-pigmentation, the micro-pigment must be concentrated at the implantation spot. We confirmed that the diffusion ring surrounding each microdot was small, with no significant changes up to 28 days post-implantation, suggesting a highly concentrated localization of the micro-pigments at each spot (Figure 4d). In the present study, the geometry of the micro-pigment-encapsulated layer was fixed; however, by adjusting the volume of the micro-pigment droplet during the fabrication process, we could further reduce the dimension of the microdots. It should be noted that to achieve a highly maintained micro-pigmentation, the target insertion depth must be below the epithelial-dermal junction. 

Implantation of PBMs creates micro-wounds that may result in skin exposure to pathogens and could affect the barrier function of skin. Thus, it is crucial to ensure that the micro-wounds heal post-implantation. To evaluate the influence of PBMs on the barrier function of the skin, the vapor flux of mice was measured using TEWL analysis up to 24 h post-implantation. Before implantation, the mean TEWL values in the PBM and control groups were approximately the same at 14.35 ± 1.94 g/m^2^ h^−1^ and 15.71 ± 1.47 g/m^2^ h^−1^, respectively. Implantation of PBMs, however, significantly increased the TEWL to 23.15 ± 2.78 g/m^2^ h^−1^. After 1 h, the vapor flux declined up to 16.47 ± 2.09 g/m^2^ h^−1^ and reached the baseline value at 2 h post-implantation (Figure 4e). The TEWL analysis confirmed that the micro-wounds created upon the implantation of PBMs were completely healed, and the skin barrier functionality was recovered to its normal state. HA is capable of accelerating the wound healing process; therefore, we assumed that the polymeric backbone matrix of PBMs played a role in the rapid skin recovery [38]. Overall, the in vivo evaluations confirmed that the PBMs are a simple, minimally invasive, and effective micro-pigmentation platform. Contrastingly, a large body of evidence suggests that the wound healing process upregulates β-catenin levels, which are known to induce hair regrowth [25,39,40]. Although further examinations are underway to confirm the effects of PBMs on hair growth at the molecular level, we expect that the micro-wounds created inside the skin would be capable of upregulating β-catenin levels, and thus might induce hair regrowth. 

Human skin has different structural properties compared with that of mouse skin; therefore, further evaluations should be directed toward confirming the efficiency of PBM via clinical trials. Moreover, to improve the hair regrowth capability of PBMs, the polymeric backbone matrix could be encapsulated with sustained release hair loss-treating agents, which we will address in a future study. 

## 4. Conclusions

In the present study, a minimally invasive micro-pigmentation platform capable of accurate implantation of a precise pigment dose into the skin was developed. Through a series of detailed evaluations, we confirmed that the intensity and dimension of 1000 μm-long PBMs was highly maintained in pig cadaver skin up to 28 days. The high concentration of micro-pigmentation over time confirmed the localization of micro-pigments at the epithelial-dermal junction. Therefore, in accordance with previous reports, we expect that the micro-pigmentation effect of PBMs will last for at least a year post-implantation. Taken together, the results of the present study suggest that the simple yet highly effective PBMs have a remarkable potential as a safe micro-pigmentation platform for individuals suffering from hair loss.

## Supplementary Materials

The following are available online at https://www.mdpi.com/1999-4923/11/11/549/s1; Figure S1. Process of pigmentation area quantification. (a) The main pigmentation area refers to the center of micro-pigmentation where the intensity is the highest. The diffusion ring is the faded area surrounding the main pigmentation area. (b) While the main pigmentation intensity was set as 90% to 100%, the intensity of diffusion ring was set as 10% to 90%. (c) The quantification was done by entering the pigmentation intensity ranges into ImageJ software and an automatic measurement of areas. Briefly, the actual image of micro-pigmentation was loaded into the software, followed by a quantification of the main pigmentation and diffusion ring areas by setting the pigmentation intensity at 90% to 100% and 10% to 90%, respectively. The resulting overlap image confirmed the accuracy of selected areas compared with the actual image; Figure S2. Schematic illustration of micro-pigment encapsulated biodegradable microneedle (PBM) micro-pigmentation patterns. To evaluate the dissolution and permeation pattern of PBMs, they were fabricated in 5 × 5 arrays with 1.5 mm pitch between each PBM. However, the array can be arranged in random manner based on the hair intensity and hair growth pattern of individuals. The density of PBMs can be also adjusted to low or high, based on the hair density of the patient; Figure S3. Fluorescence-activated cell sorting (FACS) analysis of hyaluronic acid (HA) and pigment. (a) Results indicating 97% of viable cells upon treatment with HA. (b) Pigment-treated cells exhibited a 96.9% viable cells, confirming the high biocompatibility of the pigment; Figure S4. Fracture force analysis of micro-pigment-encapsulated biodegradable microneedles (PBMs). (a) 800 μm-long PBMs showed an average fracture force of 0.55 ± 0.01 N at a displacement of 0.73 ± 0.04 mm. (b) The fracture force of 1000 μm-long PBM occurred upon applying 0.52 ± 0.02 N at a displacement of 0.91 ± 0.02 mm. Data are expressed as the mean ± SEM; Figure S5. Skin penetration force analysis. (a) The skin was pierced upon applying 0.40 ± 0.01 N by a single 800 μm-long micro-pigment encapsulated biodegradable microneedle (PBM) at a displacement of 0.38 ± 0.01 mm. (b) 1000 μm-long PBMs could penetrate the skin by applying a minimum force of 0.44 ± 0.02 N at a displacement of 0.41 ± 0.07 mm. The skin poses a different stiffness property at each point; therefore, there was a large gap between the penetration force and the displacement of the PBMs. These data, however, suggested that both PBMs were capable of successfully penetrating the skin without breakage. Data are expressed as the mean ± SEM.
