# Supplementary material for: Scalp Micro-Pigmentation via Transcutaneous Implantation of Flexible Tissue Interlocking Biodegradable Microneedles
Source: Pharmaceutics. 2019 Oct 23;11(11):549. doi: 10.3390/pharmaceutics11110549 (PMC6920983; doi:10.3390/pharmaceutics11110549)
Supplement: Supplementary file 1 [file pharmaceutics-11-00549-s001.pdf]

pharmaceutics
MDPI

Supplementary Materials: Scalp Micro-Pigmentation via Transcutaneous Implantation of Flexible Tissue Interlocking Biodegradable Microneedles

Shayan Fakhraei Lahiji, Daniel Junmin Um, Youseong Kim, Jeesu Jang, Huisuk Yang, and Hyungil Jung

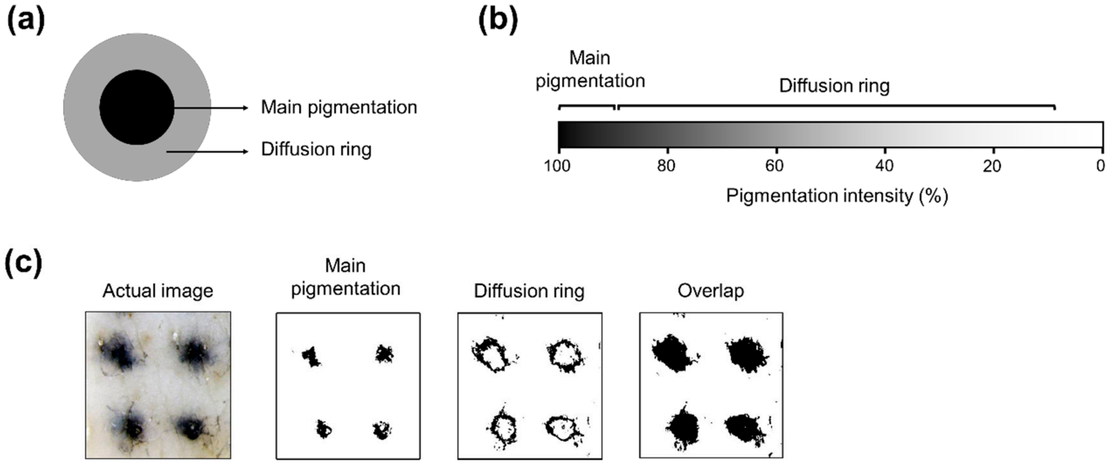

Figure S1. Process of pigmentation area quantification. (a) The main pigmentation area refers to the center of micro-pigmentation where the intensity is the highest. The diffusion ring is the faded area surrounding the main pigmentation area. (b) While the main pigmentation intensity was set as 90% to 100%, the intensity of diffusion ring was set as 10% to 90%. (c) The quantification was done by entering the pigmentation intensity ranges into ImageJ software and automatic measurement of areas. Briefly, the actual image of micro-pigmentation was loaded into the software followed by quantification of main pigmentation and diffusion ring areas by setting the pigmentation intensity at 90% to 100% and 10% to 90%, respectively. The resulting overlap image confirmed accuracy of selected areas compared with the actual image.

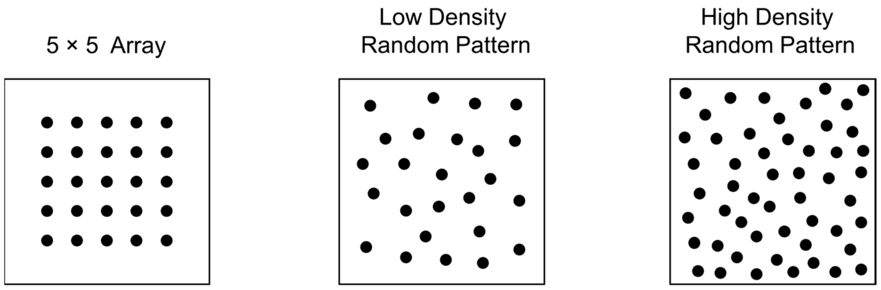

Figure S2. Schematic illustration of micro-pigment encapsulated biodegradable microneedle (PBM) micro-pigmentation patterns. To evaluate the dissolution and permeation pattern of PBMs, they were fabricated in 5 × 5 arrays with 1.5 mm pitch between each PBM. However, the array can be arranged in random manner based on the hair intensity and hair growth pattern of individuals. The density of PBMs can be also adjusted to low or high based on the hair density of the patient.

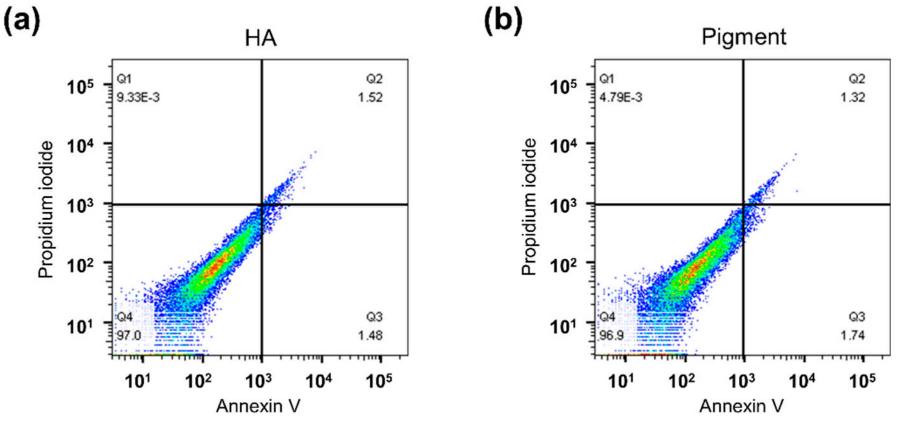

Figure S3. Fluorescence activated cell sorting (FACS) analysis of hyaluronic acid (HA) and pigment. (a) Results indicating 97% of viable cells upon treatment with HA. (b) Pigment treated cells exhibited a 96.9% viable cells, confirming the high biocompatibility of the pigment.

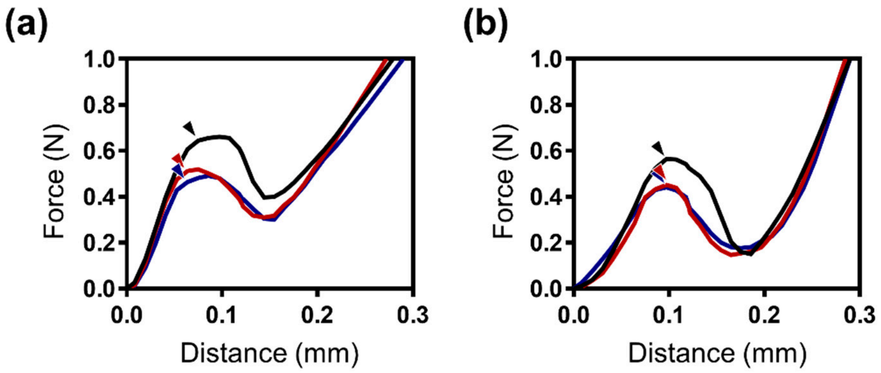

Figure S4. Fracture force analysis of micro-pigment encapsulated biodegradable microneedles (PBMs). (a) 800 μm-long PBMs showed an average fracture force of 0.55 ± 0.01 N at a displacement of 0.73 ± 0.04 mm. (b) The fracture force of 1000 μm-long PBM occurred upon applying 0.52 ± 0.02 N at a displacement of 0.91 ± 0.02 mm. Data are expressed as the mean ± SEM.

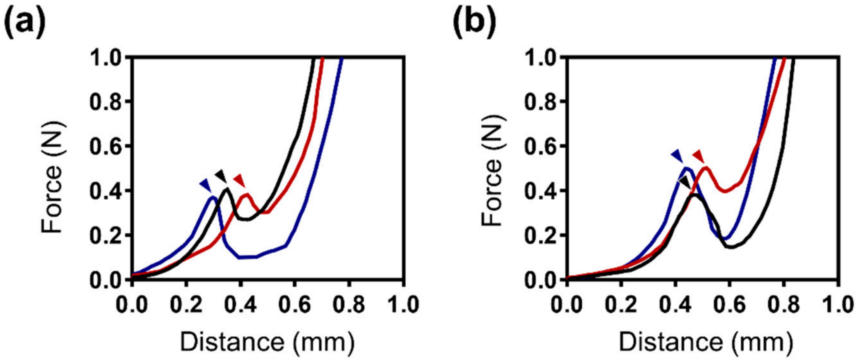

Figure S5. Skin penetration force analysis. (a) The skin was pierced upon applying 0.40 ± 0.01 N by a single 800 μm-long micro-pigment encapsulated biodegradable microneedle (PBM) at a displacement of 0.38 ± 0.01 mm. (b) 1000 μm-long PBMs could penetrate the skin by applying a minimum force of 0.44 ± 0.02 N at a displacement of 0.41 ± 0.07 mm. The skin poses a different stiffness property at each point; therefore, there was a large gap between the penetration force and the displacement of the PBMs. These data, however, suggested that both PBMs were capable of successfully penetrating the skin without breakage. Data are expressed as the mean ± SEM.
